# Influence of Material Structure on Forces Measured during Abrasive Waterjet (AWJ) Machining

**DOI:** 10.3390/ma13173878

**Published:** 2020-09-02

**Authors:** Libor M. Hlaváč, Adam Štefek, Martin Tyč, Daniel Krajcarz

**Affiliations:** 1 Department of Applied Physics, Faculty of Electrical Engineering and Computer Science, VSB–Technical University of Ostrava, 17. listopadu 2172/15, 70800 Ostrava–Poruba, Czech Republic; adam.stefek.st@vsb.cz (A.Š.); martin.tyc.st@vsb.cz (M.T.); 2 Department of Materials Science and Materials Technology, Faculty of Mechatronics and Mechanical Engineering, Kielce University of Technology, al. Tysiąclecia Państwa Polskiego 7, 25-314 Kielce, Poland; d.krajcarz@wp.pl

**Keywords:** abrasive waterjet, machining, traverse speed, material structure, material properties, cutting force, deformation force

## Abstract

Material structure is one of the important factors influencing abrasive waterjet (AWJ) machining efficiency and quality. The force measurements were performed on samples prepared from two very similar steels with different thicknesses and heat treatment. The samples were austenitized at 850 °C, quenched in polymer and tempered at various temperatures between 20 °C and 640 °C. The resulting states of material substantially differed in strength and hardness. Therefore, samples prepared from these material states are ideal for testing of material response to AWJ. The force measurements were chosen to test the possible influence of material structure on the material response to the AWJ impact. The results show that differences in material structure and respective material properties influence the limit traverse speed. The cutting to deformation force ratio seems to be a function of relative traverse speed independently on material structure.

## 1. Introduction

Abrasive waterjet (AWJ) is a tool penetrating to machining technologies for 40 years demonstrated by Natarajan et al. in their brief review [1]. AWJ is applied in many manufacturing processes. Classical machining technics like cutting and milling are studied, e.g., by Axinte et al. [2] or Rabani et al. [3]. Turning is one of the unusual machining processes and it was studied by Zohourkari et al. [4]. Even less usual application studied by Schwartzentruber and Papini is piercing [5]. Grinding was studied by Liang et al. [6] or polishing, being studied by Loc and Shiou, are rarely used applications [7], but more widespread use of these new kinds of AWJ machining is anticipated in the near future. The AWJ is capable of machining almost every type of material, independently of its thickness, if the jet energy is sufficient for penetration through material. The accuracy of this technology is closely related to either regression or theoretical models, because no complex model for all types of materials has been prepared to date. Two basic groups of models are used in practice; the first are more suitable for ductile materials and the second ones are more appropriate for brittle materials. The efforts of many research teams all over the world are focused on better understanding the erosion process as one of the important bases for improvement of contemporary models or the creation of new ones. A better understanding of erosion processes can also lead to the design of some new AWJ tools. Therefore, information about quality of the AWJ machining process is very important for quality control and it is obtained through many online and offline measurements. Conventional online methods seem to be inappropriate due to the harsh environment during the machining process and strong sensitivity to irregularities present in jet flow or material properties. Therefore, usable methods for online monitoring of the AWJ processes are still being sought.

Investigation of the acoustic emissions studied (e.g., by Rabani et al. [8]) is one of methods tested for the monitoring of AWJ processes. Another type of AWJ cutting process monitoring was presented by Hreha et al., studying the relations between the vibrations of cut material measured by accelerometers and the surface roughness of cut wall [9]. However, the experiences of scientists described by Mikler show that more than 25% of the information from acoustic emissions or vibrations may be incorrect due to the misinterpretation of the complicated dynamic signal [10]. The attempts to determine the quality of the machining process by vibration signals measured on cutting heads were also presented in several research articles. Fabian and Salokyová [11,12,13] presented in these articles the measurements and analyses of signals from accelerometers placed on a cutting head (mixing chamber) and attempted to determine the relationship between some resonation frequencies and material cutting quality.

Research of new monitoring and control methods continues and research activities take various forms. Karas et al. [14], for example, tried to determine AWJ characteristics through mathematical modelling in Computational Fluid Dynamics CFD methods to prepare a tool to predict manufacturing processes results. Li et al. [15] studied deformation of stainless steel 304 and its mechanical properties after AWJ processing by using several methods. Pahuja et al. investigated the behaviour of the abrasive water jet during its passage through a system of plates made from various kinds of materials [16]. Their findings are very important for certain industrial applications, namely in aerospace. However, acoustic emissions, cutting head vibrations and other dynamic methods may be influenced by the dimensions of machines, their stiffness, or the dimensions of machined pieces of materials as mentioned by Mikler [10]. Searching for new ways of measurements applicable to new technologies has been mentioned by Królczyk et al. [17]. Therefore, some alternative to dynamic measurements have been investigated.

Forces can be used both for dynamic and static measurements. One of the first AWJ force measurements has been described by Li et al. in 1989 [18]. Authors tried to measure the force response of both the pure and abrasive water jet regarding several selected parameters—stand-off distance, water nozzle diameter, focussing tube diameter, abrasive mass flow rate and abrasive size. The results are inspirational for further research, but the basic relationships correspond with expectations based on theoretical analyses and contemporary measurements. In the past, the force sensors were also used for the determination of the pure waterjet velocity profile and diameter, as presented in Vala and Vala et al. in [19,20] or for the investigation of possible safety hazards of hand operating tools described by Hlaváčová and Vondra [21]. The problems with influencing the measured signal by dimensions of machined pieces of material could be partially be reduced using a measurement on samples with equal dimensions. Therefore, the force sensor measuring x-y-z forces during AWJ cutting has been designed and patented by Mádr et al. [22]. Here, the force sensor is tested in order to find out the limits of its use for the continuous monitoring of AWJ processes and their control.

The measurements presented in this article are aimed at the role of material structure impact on measured data. During the measurements of forces on various metal samples, it was mentioned that the most important parameter changing with this kind of material is the limit traverse speed. The mutual ratio of forces in the axes seemed to be influenced just by this parameter and respective “portion” of this value represented by the “actual traverse speed”. The intent of the presented research was to eliminate the influence of other possible sources of material difference (elemental composition of the material, density). Therefore, the material enabling an extensive change of strength characteristics and thus the limit traverse speeds has been selected (in two very close modifications). Three different traverse speeds were used for testing. The experiments were performed on two similar steels with a broad scale of strength characteristics created by heat treatment of original raw materials. Therefore, the material structure and respective material characteristics differ significantly, while the density and elemental composition of material are identical. The results of force measurements confirm the influence of traverse speed on the material response against AWJ penetration, but the direct influence of material strength characteristics on the force response is not strong. On the contrary, the force measurement results indicate that the change of material properties influences the limit traverse speed of AWJ machining. This fact causes movement with identical relative traverse speeds that induce cutting forces in kerf (tangential and normal) in the same mutual ratio.

## 2. Theoretical Background

The theoretical model, used for the calculations necessary to determine the relative traverse speeds (the independent variables in this research), is based on the limit traverse speed $v_{P\lim}$ determined for each material by Hlaváč et al. [23] according to his equations. The respective jet velocity loss $\alpha_{e}$ is to be determined experimentally because of the interaction time uncertainty (explanation of this parameter and its determination was presented by Strnadel et al. in [24]). The respective equations are based on jet and material characteristic parameters:

(1) $$v_{P\lim}\;=\;{\left[\frac{C_{A}\;S_{p}\;\pi \;d_{o}\;\sqrt{2\rho_{j}p_{j}^{3}\;e^{-5\xi_{j}L}}\;(1\;-\;\alpha_{e}^{2})}{8\;H\;\left(p_{j}\rho_{m}\alpha_{e}^{2}\;e^{-2\xi_{j}L}\;+\;\sigma_{m}\rho_{j}\right)}\right]}^{\frac{2}{3}}\;-\;v_{Pmin}$$

(2) $$\alpha_{e}=1-\frac{\sqrt{{2p}_{j}^{3}}\;HVt_{i}}{8\sqrt{\rho_{j}}\;\sigma_{m}a_{m}}$$

The variables used in Equations (1) and (2) have the subsequent meaning: $v_{P\lim}$ the limit traverse speed; $C_{A}$ the coefficient modifying abrasive water jet performance according to the changing content of abrasive below “saturation level” (above this level, the jet performance increases no more and $C_{A}=1$); $S_{P}$-ratio between the quantity of non-damaged grains (i.e., not containing defects) and the total quantity of grains in the supplied abrasive material; $d_{o}$-diameter of the water nozzle (orifice); $\rho_{j}$-density of abrasive jet (conversion to homogeneous liquid); $p_{j}$-pressure obtained from Bernoulli’s equation for liquid with density and velocity of abrasive jet; $\xi_{j}$-attenuation coefficient of abrasive jet in the environment between the focusing tube outlet and the material surface (usually air); L-stand-off distance (distance between the focusing tube outlet and the material surface); $\alpha_{e}$-coefficient of abrasive water jet velocity loss in the interaction with material (experimentally determined); H-material thickness; $\rho_{m}$-density of material being machined; $\sigma_{m}$-strength of material being machined; $v_{Pmin}$-minimum limit traverse speed of cutting–correction for the zero traverse speed (usually the value $v_{Pmin}=a_{n}/60$ is used, where $a_{n}$ is the average abrasive particle size after the mixing process inside the mixing head and focussing tube); HV-material hardness; $t_{i}$-interaction time; $a_{m}$-mean size of particles (elements) of material–grains or their chips.

The declination angle of striations measured at the bottom edge of a sample wall for a certain traverse speed introduced by Hlaváč [25] can be used for the calculation of the limit value according the equation

(3) $$v_{P\lim}=v_{P}{\left(\frac{\vartheta_{\lim}}{\vartheta }\right)}^{\frac{2}{3}}$$

where the traverse speed $v_{P}$ is the experimental one for which the declination angle ϑ is measured on the kerf wall [25]. The declination angle or respective relative traverse speed (the ratio of $v_{P}$ and $v_{P\lim}$) can be utilized for determination of some quantities and process parameters regarding required quality of cutting, i.e., declination angle of striations on the kerf walls [23] and inclination of the walls (the taper) [26]. Because the traverse speed of the cutting head is the most easily controlled parameter of AWJ machining, it is very important to know the relationship between this speed and resulting quality, as it was presented by Hlaváč et al. [27]. However, the limit declination angle used for the calculation of the limit traverse speed depends on the jet energy and material parameters. If the energy of the AWJ is sufficient for full development of both types of material wear inside kerf, the limit declination angle is close to 45°. The limit declination angle value decreases, when the AWJ energy is decreasing regarding the necessary one for both types of wear, as indicated in [28]. Provided that only cutting wear can be utilized, the declination angle can decrease to 22.5° and for very hard or very thick materials the value 15° seems to be the limit one. These limits could be influenced not only by jet energy and macroscopic material properties, but also by abrasive grains rigidity and material structure.

**Figure 1:**
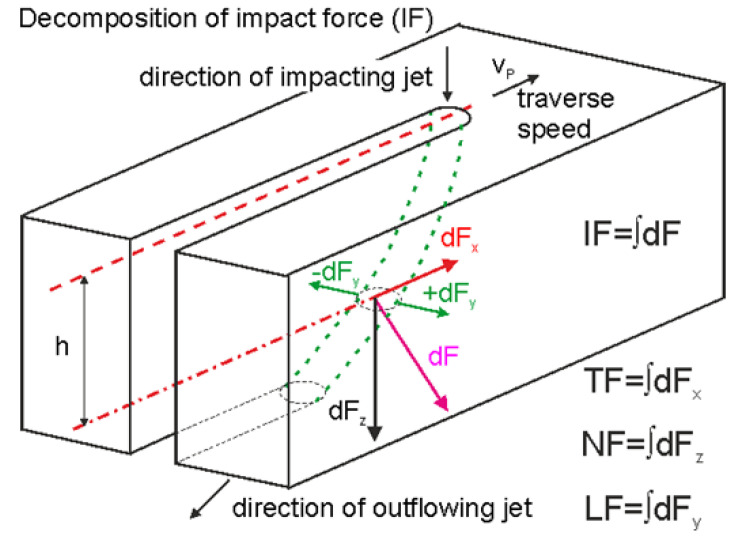
Decomposition of the total impact force acting on the kerf head into the cutting (TF), deformation (NF) and transverse (LF) forces; the forces elements are distinguished by subscripts corresponding with force sensor axes directions, measured values are integral values in the selected direction at a given moment.

The scheme of the impacting force decomposition inside the kerf into cutting forces (tangential), and the deformation (normal) and transverse (lateral) components is presented in Figure 1. Elements of the components are signed by the axes of the force sensor.

Considering the force decomposition, the following is presumed of this behaviour of new quantities: “The cutting to deformation force ratio (CDFR) depends on the ratio of the cutting-to-deformation wear in the produced kerf”. It is anticipated that an increase of the traverse speed from a zero value to the limit causes an increase of the CDFR up to a certain maximum value, achieved approximately for a half of the limit traverse speed. This maximum corresponds with the maximum portion of cutting force. Further increase of the traverse speed causes further increase of the deformation force. This is caused by the increasing portion of the deformation wear due to kerf head curvature, while the cutting wear cannot increase more. Therefore, the CDFR decreases for traverse speeds higher than a half of the limit traverse speed. This consideration is valid only in cases when the energy of the jet is sufficient for the full development of both types of wear. Lower energies than necessary (usually during the cutting of very thick materials) makes it so that only cutting wear can be used to penetrate through material. The residual jet energy inside the deep kerfs is insufficient for the proper development of the deformation wear and it reflects back from the kerf as a reverse flow when the conditions for cutting force are exceeded. This situation occurs when the outlet declination angle overcomes a value of approximately 22.5° for medium-thick materials (1.5–2.5 thicker than appropriate to jet energy) or 15° for very thick materials (for thickness 2.5 higher than the appropriate one).

The theoretical model presented in [23,24,25,26] makes it possible to limit the transfer values determined for one machining configuration to another, reducing the necessary experimental work. Equation (4) is based on functional dependences of traverse speed on respective changing variables-Equations (1) and (2). Therefore, the values ones determined for certain machine configuration like, e.g., in [27], need not be determined again making new samples and measuring respective angles on them. Provided that the nozzle diameter, pressure and abrasive type are changed, the limit traverse speed for configuration 2 is calculated from the value for configuration 1 through the equation

(4) $$v_{P\lim2}=\eta_{A21}\frac{d_{2}}{d_{1}}\sqrt{\frac{p_{2}^{3}}{p_{1}^{3}}}v_{P\lim1}$$

where $v_{P\lim2}$ is the limit traverse speed calculated for machine configuration 2; $v_{P\lim1}$ is the limit traverse speed already known for machine configuration 1; $\eta_{A21}$ is a ratio of abrasive qualities (the second to the first one), if they differ; $d_{1},\;d_{2}$ are the respective nozzle diameters of configurations; $p_{1,\;}p_{2}$ are the respective pumping pressures of configurations. The relative traverse speed (the main quality indicator) is then determined as

(5) $$v_{R}=\frac{v_{P}}{v_{P\lim}}$$

To make the research as objective as possible, the experiments were performed on a different device than the one originally used to determine the limit traverse speeds and the first cutting studies. The limit traverse speeds were calculated from original values using Equation (4). The ratio of cutting and deformation forces were determined and it its dependence was studied on relative traverse speed to approve its relationship with material structure, because limit traverse speeds differ for individual samples due to the differing structures of the materials (see [24] for details).

## 3. Materials and Methods

Several metal samples with different structures and respective strength characteristics were used for experiments. The two similar steels 34CrMo4 (DIN norm) were used, differing namely by nickel ratio. The raw material for each one was in another thickness. The composition of both steels is presented in Table 1.

The samples were prepared from material austenitized at 850 °C, quenched in polymer and tempered at various temperatures; their tempering and respective uniaxial tensile strengths ($\sigma_{m}$) and Vickers hardness (*HV10*) for individual samples are summarized in Table 2.

**Figure 2:**
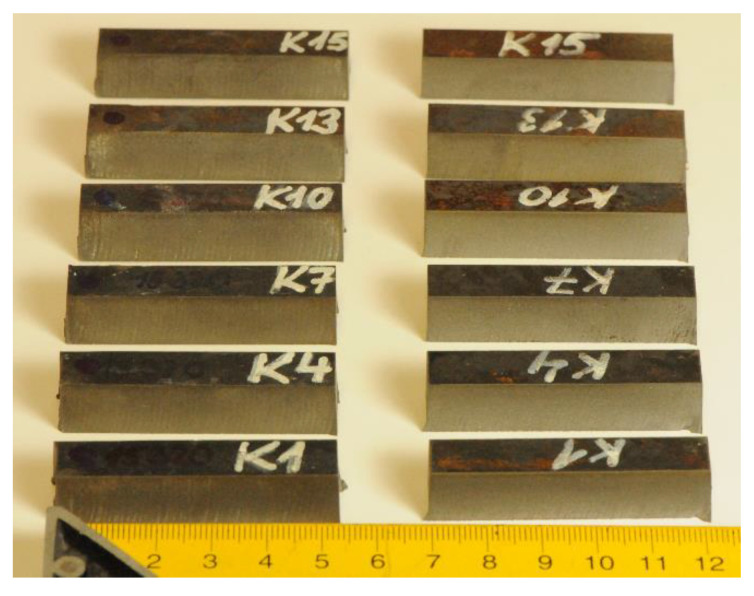
Samples used for analysis of material structure impact on declination angle (see [24]).

The samples were previously used for research presented in [24], where the influence of tempering on the material structure, namely the amount of carbides, was largely discussed. Therefore, the limit traverse speeds $v_{P\lim1}$ are determined from the original cutting parameters used for sample preparation in laboratory at the VŠB–Technical University of Ostrava (see [24]). Some of the samples analysed in [24] are presented in Figure 2.

The limit traverse speeds $v_{P\lim2}$, necessary for experiments in Kielce, are calculated from Equation (4) for respective values of parameters summarized in Table 3 (for workplace actually used for experimental work–in Kielce–and the one used for previous experiments–in Ostrava). Both limit traverse speeds ($v_{P\lim1},\;v_{P\lim2}$) are presented in Table 2 for respective samples.

All cutting experiments were performed at the Faculty of Mechatronics and Mechanical Engineering, Department of Materials Science and Materials Technology, the Kielce University of Technology, Poland. Experimental factors and parameters of the abrasive waterjet are summarized in Table 3.

**Figure 3:**
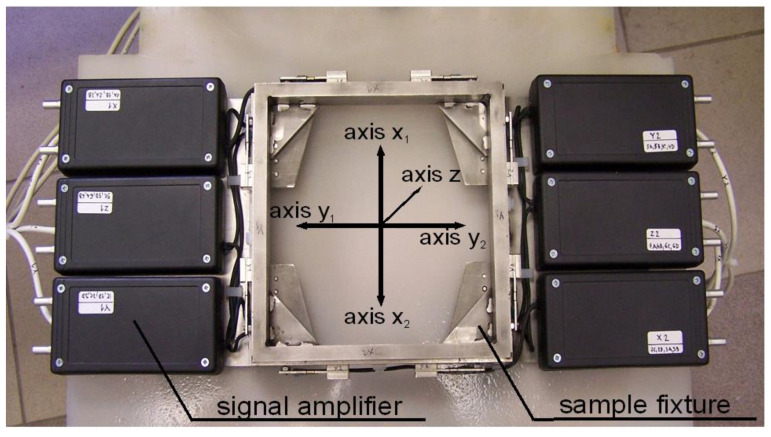
Overall view of the force sensor–black boxes contain amplifiers for strain gauges.

The cuts in steels were performed at fixed three traverse speeds for each thickness of tested steels. The respective relative traverse speeds were calculated from these three pre-set traverse speeds and the respective limit traverse speeds $v_{P\lim2}$ calculated from values $v_{P\lim1}$ listed in Table 2. The forces in x and z directions were measured by special measuring device being able to measure forces in three orthogonal axes [22]. Design of this device is shown in Figure 3.

Signals from all measuring directions were recorded to a computer via the Signal Express program. After that, a raw voltage signal was processed with a program prepared in LabVIEW. The entire measuring system contains a DC source, the measuring part with deformation elements covered by extensometers, electric signal amplifiers (Figure 3), AD transducer, and computer with recording and processing software (Signal Express, LabVIEW, Austin, TX, USA).

**Figure 4:**
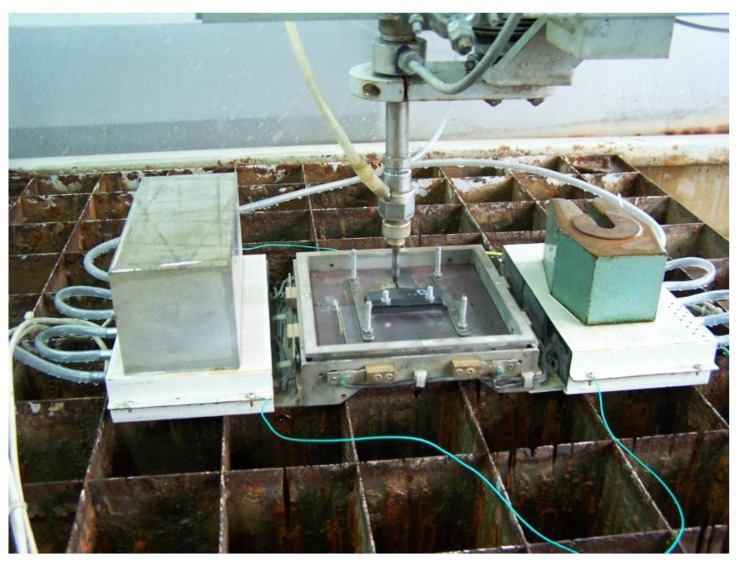
Force sensor on the cutting table in Kielce.

The overall experimental procedure starts by placing the force sensor on the cutting table and fixing it to the grid, usually through putting heavy elements on the boxes with electronics, as presented in Figure 4. The sample is mounted to the removable plate with a central hole and placed into the frame signed “sample fixture” in Figure 3. The cutting machine and measuring system are switched on and prepared for starting the cut.

**Figure 5:**
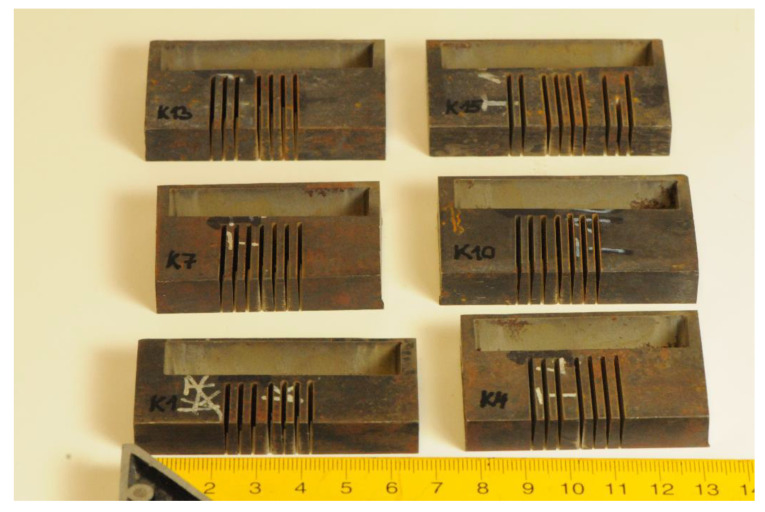
Samples after cutting for force measurement.

The recording on the measuring system is started first and then the cutting machine is put into operation. The movement of the cutting jet starts from outside the sample and ends approximately 20 mm inside the sample. The resulting shape of the samples after cutting is presented in Figure 5.

**Figure 6:**
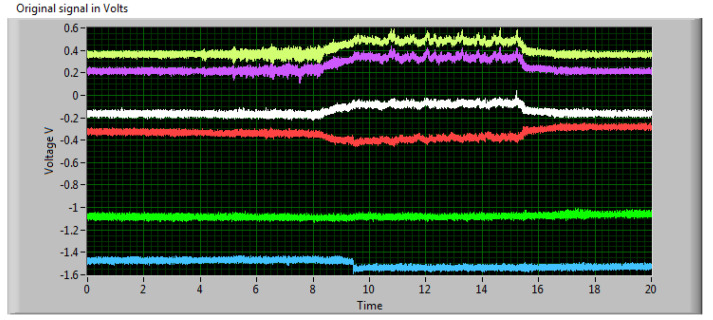
Electric signals measured by force sensor during cutting process of CK 44. Signal lines: yellow (1st from the top)–one part of the z axis, magenta (2nd from the top)–second part of the z axis, white (3rd from the top)–x^+^ axis, red (4th from the top)–x^−^ axis, green (5th from the top)–y^+^ axis, blue (the bottom line)–y^−^ axis.

**Figure 7:**
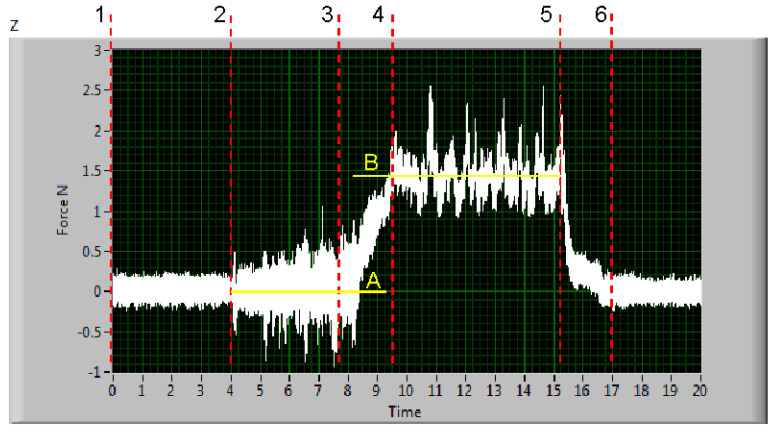
Force signal in *z*-axis after processing in LabVIEW program. Description of force signal parts: 1–2–signal before starting of the cutting process; 2–3–signal for the jet moving towards material (it is just impacting water in the waste energy attenuating vessel); 3–4–signal for jet starting to penetrate to material from the open side; 4–5–signal from the cutting through material; 5–6–signal from ending the cutting and stopping the machine; 6 to the end–signal from non-cutting system; A and B–lower and upper levels of force: the difference is force value in *z*-axis.

A typical measured signal is shown in Figure 6. There are electric signals from all Wheatstone bridges formed by extensometers placed on deformation elements of the force sensor. One bridge is for the x^+^ axis (two deformation elements), the next one for x^−^ axis (two deformation elements). Two more bridges are for y^+^ and y^−^ axes. The force in the selected direction is a mean of the absolute values in both directions of the respective axis. It was a different situation with z axis, because only the direction towards the ground is relevant. There are also two couples of deformation elements with respective bridges, but each of them registers only a part of the total force in the z direction. Therefore, the resulting force is a sum of these parts. The force signal after processing in LabVIEW program is presented in Figure 7.

## 4. Results

**Figure 8:**
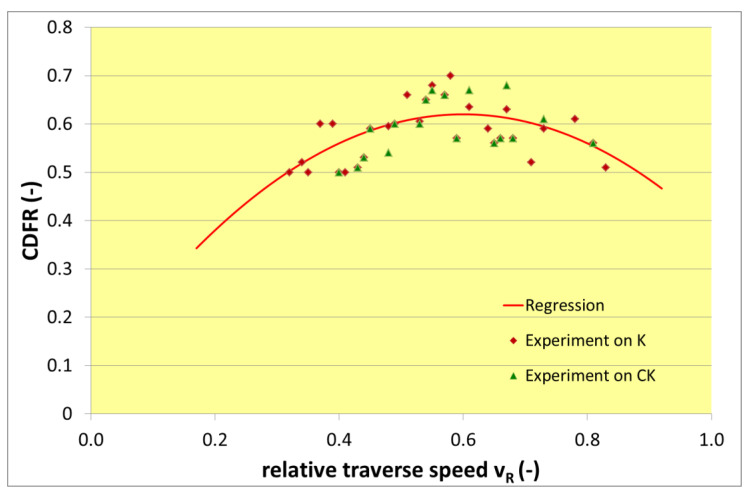
Graph of relationship between relative traverse speed and CDFR.

The forces in the x-direction and z-direction (cutting and deformation ones respectively) were calculated from measured signals in the self-prepared program for signals processing in the LabVIEW™. The respective force values are the differences of the mean values of the signals in parts 4–5 and 2–3 (see Figure 7). The levels of average forces are represented by lines A (lower level) and B (upper level). The difference of these two values is the force value of the respective axis in a particular measurement. The relative traverse speeds were calculated from Equation (5) from the actual used traverse speed (the heading line of Table 4) and the respective limit traverse speed $v_{P\lim2}$ (see Table 2) calculated for each material from $v_{P\lim1}$ through Equation (4). CDFR values for various relative traverse speeds are summarized in Table 4 and graphically presented in Figure 8.

It can be seen that parabolic function is a good approximation of relationship between relative traverse speed and CDFR (representing cutting to deformation wear ratio inside the produced kerf). Nevertheless, no evident relationship on material structure is detected in the CDFR primarily. Quite the opposite—it seems like the CDFR is identical for a certain relative traverse speed independently of the material structure.

The respective regression equation for the calculation of the CDFR value from respective relative traverse speed value was determined in Excel for all measured values as.

(6) $$CDFR=-1.5\;v_{R}^{2}+1.8\;v_{R}+0.08$$

Points (experimental results) presented in Figure 8 are determined as ratios from forces measured in x and z directions for respective relative traverse speeds (see Table 4). The regression equation and the respective curve in Figure 8 are presented just as a partial proof of the hypothesis presented in the theoretical portion and stating that the CDFR increases with increasing traverse speed up to the maximum at around the half of the limit traverse speed, and then it decreases.

## 5. Discussion

All experimental results indicate that material structure influences the material response to AWJ namely through changes of the limit traverse speeds. Material strength, hardness and structure, namely the amount and size of carbides [24], cause differences in absolute value of the limit traverse speed. This factor, the limit traverse speed, seems to be the main AWJ characteristic influenced by material structure directly, and probably the only one. The presented research was aimed at testing the influence of the material structure on the forces measured during AWJ machining, namely cutting. Therefore, two steels with a large change of structure depending on heat treatment were selected for tests. Each of the steels was of a different thickness. Heat treated samples with identical thickness were cut using the same scale of three traverse speeds. The different steel structures cause different limit traverse speeds. Therefore, it is evident that the ratio of the actual and limit traverse speed (relative traverse speed) should be the most appropriate independent variable for evaluation of the structure influence on the CDFR.

According to Hlaváč’s theoretical model represented by Equations (1)–(3), it was anticipated that the identical relative traverse speeds produce similar cutting to deformation force ratios indicating so analogical ratios of cutting to deformation wear inside the produced kerfs. Differences in the CDFR are caused by a change of the relative traverse speed due to either the uncertainty of the material properties or the AWJ characteristics. Experimental results show independence of the CDFR on material thickness for AWJ energies sufficient for the full development of both types of material wear inside the produced kerf.

The contemporary research can be supported by conclusions based on results presented in [24]. The most important experimental and calculated results regarding size and volume percentage of carbides are summarized together with the respective limit traverse speeds in Table 5. Equation (7) for the calculation of carbide sizes $d_{c}$ regarding the tempering temperature $T_{t}$ has been determined from experimental results partially published in [24]

(7) $$d_{c}=0.0000004T_{t}^{3}-0.0003787T_{t}^{2}+0.1202T_{t}+15$$

The description of experimental materials presented in [24] states: “The increase in density of fine carbides at higher tempering temperatures is caused by the coarsening of carbides accompanied by a change in their coherence to non-coherent phase boundaries. The plastic deformation is easier in the presence of coarse carbides, primarily because dislocations may overcome fields of coarse carbides during plastic deformation by means of the Orowan mechanism, which requires relatively low energy [29]. The significant effect of fine coherent precipitates on hardening is replaced by hardening by means of non-coherent carbides, in which increasing carbide size is accompanied by a decline in hardening [30].” Therefore, it was necessary to test if the changing structure of material has the direct influence on the CDFR trends.

**Figure 9:**
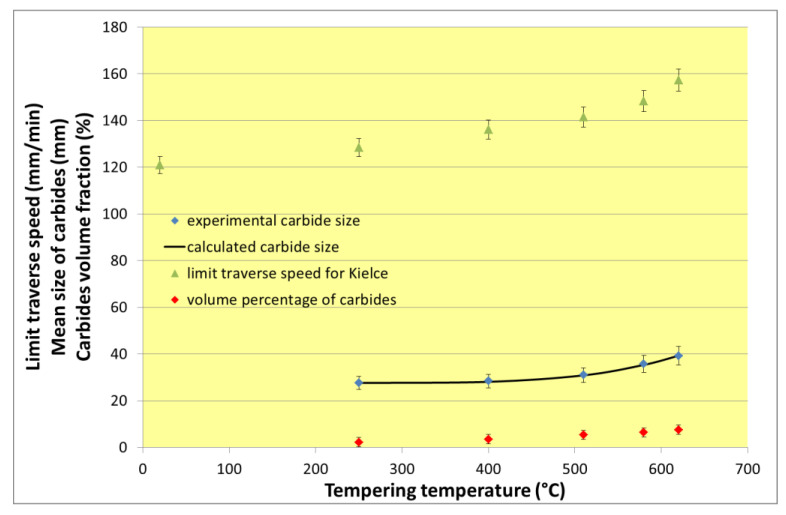
Trends of dependences of the carbide sizes, volume percentage of carbides and the limit traverse speeds on the tempering temperatures of samples used for research of material structure influence on AWJ cutting forces (the uncertainty magnitudes of experimental and calculated values are added).

Figure 9 is showing a comparison of trends of the carbide size, volume percentage of carbides and the limit traverse speed dependence on tempering temperature. All three trends are identical, proving that the limit traverse speed clearly depends on the structure of the experimental material. Simultaneously, the difference between these trends and the trend of the CDFR dependence on the relative traverse speed confirms that the limit traverse speed is the proper variable influenced by the material structure. The CDFR is almost independent on the material structure, because it has a similar trend and values for similar or identical relative traverse speeds (portions of the respective limit traverse speed) independently on the absolute value of the limit traverse speed.

Another way of explanation is based on the detail description of the cutting process with increasing traverse speed. When the traverse speed is close to zero, a very small part of the impacting jet is cutting material, i.e., the force acting towards the traverse speed direction (the tangential force) is small. Simultaneously, the part of the jet cross-section circumference projected to the plane perpendicular to the traverse speed is just a jet diameter. The force in the direction of jet flow (the normal one) is also small, but higher than cutting one due to the greater part of jet acting on the jet border (one half of the jet cross-section circumference, i.e., π/2 times longer than for the cutting force). By increasing the traverse speed the jet delay in the kerf makes the forehead of the kerf curve, increasing with the deformation wear of material. Simultaneously, the pushing of the jet towards traverse speed direction also increases. Up to a certain traverse speed, the tangential force pushing on the kerf forehead increases more rapidly than normal force increasing due to kerf forehead curving. However, for a certain traverse speed, the curvature of the kerf forehead is so high that tangential force cannot increase more, because part of the force pushing towards traverse speed direction projects to the normal direction. Therefore, the ratio of cutting to the deformation force decreases. This whole scale of ratios can be observed when the energy of the jet is sufficient for a full development of both wear types in the kerf. In the case of very thick material cutting, when only the cutting wear can be utilized, the CDFR relationship on the relative traverse speed either ends in the maximum value or at some point of the increasing part of the curve. This knowledge can be used for AWJ machining monitoring and control.

## 6. Conclusions

The subsequent conclusions resulting from presented research work were found out during the evaluation of data measured by the force sensor on the steel with a large scale of strength parameters prepared by various thermal processing of identical raw materials:

(1) Changes in material structure influence primarily the limit AWJ traverse speed. This finding is consistent with previously observed relationships between the material structure and the limit traverse speed published in prior articles. The benefit of the presented research is that relationship is confirmed independently on material composition and density.
(2) Differences in limit traverse speed caused by structure changes inflict different relative traverse speeds for the fixed testing traverse speed set. Therefore, it is possible to evaluate a large set of experimental results with a limited number of samples to yield broad range of relative traverse speeds. This is an important consequence of the application of the presented theoretical base for experimental planning and design.
(3) Dependence of CDFR on relative traverse speed has a parabolic relationship and the maximum is located near the value $0.5\;v_{P\lim}$. This finding is one of the important new results of the presented research. It is confirmed on steel samples 6 mm and 10 mm thick and AWJ power density higher than 8 GW·m^−2^.
(4) The parabolic shape of the functional relationship between relative traverse speed and the CDFR is confirmed for AWJ characteristics being sufficient for a full development of both wear types (cutting and deformation ones) inside the produced kerf. An increase of material thickness or decrease of AWJ power may cause that only the cutting wear can be present and the deformation wear is either substantially suppressed or fully eliminated. Searching for respective limits is the aim of subsequent research.
(5) It can be anticipated that decreasing of the limit traverse speed for increasing material thickness causes shift of the CDFR maximum closer to the limit traverse speed, i.e., the maximum is either shifted to the relative traverse speed equal to one or no maximum occurs at all (the curve of CDFR relation on relative traverse speed has only increasing character).
(6) The dependence of the CDFR primarily on relative traverse speed and independence of the CDFR on the material structure are the most important new findings of presented research, because they make force measurements usable for monitoring and control of all kinds of materials, provided that AWJ power is sufficient for both kinds of material wear inside kerf.

## Figures and Tables

**Table 1:** Chemical composition of two modifications of 34CrMo4 steel in weight %, hydrogen content is in ppm.

Steel Kind	C	Mn	Si	Cr	Ni	Mo	Cu	V	Al	P	S	N	H
K	0.37	0.84	0.26	1.15	0.03	0.208	0.02	0.074	0.0113	0.012	0.004	0.0113	1.3
CK	0.36	0.84	0.29	1.13	0.24	0.200	0.04	0.093	0.0270	0.011	0.003	0.0120	1.1

**Table 2:** Steel 34CrMo4 samples austenitized at 850 °C, quenched in polymer and tempered at various presented temperatures.

Sample Mark (10 mm Thick)	Tempering Temperature (°C)	Uniaxial Strength (MPa)	Vickers Hardness HV 10	Limit Traverse Speed $v_{P\lim1}$ (mm/min)	Limit Traverse Speed $v_{P\lim2}$ (mm/min)
K37	20	2230	589	180	121
K38	250	1865	521	192	129
K39	400	1560	459	203	136
K40	510	1340	391	211	141
K41	580	1190	363	222	148
K42	620	1060	330	235	157
**Sample Mark (6 mm Thick)**	**Tempering Temperature (°C)**	**Uniaxial Strength (MPa)**	**Vickers Hardness HV 10**	**Limit Traverse Speed $v_{P\lim1}$** **(mm/min)**	**Limit Traverse Speed $v_{P\lim2}$** **(mm/min)**
CK43	20	2160	581	278	186
CK44	250	1860	514	306	204
CK45	400	1550	464	330	221
CK46	510	1320	405	339	227
CK47	580	1230	372	341	228
CK48	640	970	314	378	253

**Table 3:** Summary of factors and parameters used for experiments in Kielce and the values used for determination of the original limit traverse speeds $v_{P\lim1}$ in Ostrava (in brackets).

Variable (Unit)	Value
Pump pressure (MPa)	250 (380)
Water orifice diameter (mm)	0.33 (0.25)
Focusing tube diameter (mm)	1.02 (1.02)
Focusing tube length (mm)	76 (76)
Abrasive mass flow rate (g/min)	240 (240)
Abrasive material average grain size (mm)	0.177 (0.180)
Abrasive material type	Indian garnet (Australian garnet)
Abrasive quality ratio $\eta_{A21}$	0.95
Stand-off distance (mm)	2 (2)
Traverse speed (mm/min)	50–150

**Table 4:** CDFR values and the respective relative traverse speeds $v_{R}$ (in brackets).

Samples 34CrMo4/10 mm	50 mm/min	75 mm/min	100 mm/min
K37	0.50 [0.41]	0.60 [0.62]	0.51 [0.83]
K38	0.60 [0.39]	0.70 [0.58]	0.61 [0.78]
K39	0.60 [0.37]	0.69 [0.55]	0.57 [0.73]
K40	0.50 [0.35]	0.61 [0.53]	0.52 [0.71]
K41	0.52 [0.34]	0.66 [0.51]	0.58 [0.67]
K42	0.50 [0.32]	0.65 [0.48]	0.59 [0.64]
**Samples 34CrMo4/6 mm**	**100 mm/min**	**125 mm/min**	**150 mm/min**
CK 43	0.60 [0.53]	0.68 [0.67]	0.56 [0.81]
CK 44	0.54 [0.48]	0.67 [0.61]	0.61 [0.73]
CK 45	0.59 [0.45]	0.66 [0.57]	0.57 [0.68]
CK 46	0.53 [0.44]	0.67 [0.55]	0.57 [0.66]
CK 47	0.51 [0.43]	0.65 [0.54]	0.56 [0.65]
CK 48	0.50 [0.40]	0.60 [0.49]	0.57 [0.59]

**Table 5:** Tempering temperatures of the 34CrMo4 steel, respective experimental carbide sizes, carbide sizes calculated from experimentally based Equation (7), volume percentage of carbides and the limit traverse speeds set for the cutting machine in Kielce.

Sample Mark (10 mm Thick)	Tempering Temperature (°C)	Measured Size of Carbides d_c_ (m)	Calculated Carbide Size d_c_ (m)	Percentage of Carbides (%)	Limit Traverse Speed (mm/min)
K37	20				121
K38	250	27.54	27.63	2.20	129
K39	400	28.36	28.09	3.50	136
K40	510	31.01	30.86	5.37	141
K41	580	35.72	35.37	6.42	148
K42	620	39.26	39.28	7.53	157
